# The Adaptive and Innate Immune Cell Landscape of Uterine Leiomyosarcomas

**DOI:** 10.1038/s41598-020-57627-1

**Published:** 2020-01-20

**Authors:** Marco Manzoni, Maddalena M. Bolognesi, Asier Antoranz, Rosanna Mancari, Silvestro Carinelli, Mario Faretta, Francesca M. Bosisio, Giorgio Cattoretti

**Affiliations:** 1 Pathology, Department of Medicine and Surgery, Università di Milano-Bicocca, Via Cadore 48, Monza, (MI) Italy; 2 National Technical University of Athens, Zografou Campus, 9 Iroon Polytechniou str., 15780 Zografou Athens, Greece; 3 Division of Gynecologic Oncology and Pathology, European Institute of Oncology, Via Ripamonti 435, 20141 Milan, Italy; 4 Department of Experimental Oncology, European Institute of Oncology, IRCCS, via Adamello 16, 20139 Milan, Italy; 5 Laboratory of Translational Cell and Tissue Research, KU Leuven, Herestraat 49, 3000 Leuven, Belgium

**Keywords:** Tumour immunology, Oncology

## Abstract

Reactivation of the anti-tumor response has shown substantial progress in aggressive tumors such as melanoma and lung cancer. Data on less common histotypes are scanty. Immune checkpoint inhibitor therapy has been applied to few cases of uterine leiomyosarcomas, of which the immune cell composition was not examined in detail. We analyzed the inflammatory infiltrate of 21 such cases in high-dimensional, single cell phenotyping on routinely processed tissue. T-lymphoid cells displayed a composite phenotype common to all tumors, suggestive of antigen-exposure, acute and chronic exhaustion. To the contrary, myelomonocytic cells had case-specific individual combinations of phenotypes and subsets. We identified five distinct monocyte-macrophage cell types, some not described before, bearing immunosuppressive molecules (TIM3, B7H3, VISTA, PD1, PDL1). Detailed *in situ* analysis of routinely processed tissue yields comprehensive information about the immune status of sarcomas. The method employed provides equivalent information to extractive single-cell technology, with spatial contexture and a modest investment.

## Introduction

The adaptive immune system has evolved into a very refined and complex coordination of multiple actors (T, B, dendritic, NK cells etc.) devoted to the control of exogenous attackers, while avoiding the collateral damage of the self^1^. The onset of cancer affects both the adaptive and the innate immune system via multiple mechanisms: by increasing the cell turnover and mass, by creating new vascularized tissue, by presenting new antigens in a previously tolerant scenario and by recruiting heterotopic inflammatory cells^2^.

How the adaptive immune system deals with cancer has recently gained attention because of the promising clinical results with personalized medicine targeting the T-cell response against tumors^3,4^. The immune reaction against the tumor is placed in check by the combined action of tumor escape and naturally occurring mechanisms which dampen the immune response^3–8^. These mechanism may co-exist and function independently^9^ and the result is a cancer immunogram in which each tumor has a combination of the various components^4,7^. Inhibition of blocking immune checkpoints via therapeutic antibodies restores a pre-existing anti-tumor T-cell response and results in prolonged remission or cure of otherwise lethal cancers^10–12^.

Not all cancer patients respond to a checkpoint inhibitor therapy. The tumor mutational burden, i.e. the ability to present neoantigens to the adaptive immune system, has been identified as one major biomarker predictive of response^10,12^. Hypermutating tumors are good candidates for this therapy^13^. An altered DNA copy number or ongoing DNA damage repair mechanisms^14,15^ may also recruit intratumoral lymphocytes (TILs).

Distinctive modules of inflammation shared by diverse cancer histotypes have been revealed by pan-cancer analysis of deposited gene-expression databases^16,17^: these studies have shown that tumor mutation burden and CD8 TIL infiltration have an impact on the prognosis, but a macrophage signature may also affect the outcome^17^. Some studies have identified a macrophage signature independent of tumor type^18^, yet the awareness of the complex regulation of macrophage biology may suggest otherwise^19^.

Leiomyosarcomas originating from myometrium (ULMs), have not been intensively investigated because of their low incidence and because they are perceived as a minor target for immune intervention, given the few tumor TILs on H&E sections and the low mutational burden^20^.

As few as two dozens ULMs have been treated with checkpoint inhibitors, with dismal results^21–23^. However, with the exception of one responsive case^22^, in none of them the TILs or the macrophages have been thoroughly studied.

Here we present a comprehensive high-dimensional analysis of the inflammatory infiltrate in 21 sarcoma cases with a panel of 40 markers including lineage specific leukocyte proteins, activation markers and component of the immunological synapse, by using a novel robust method, effective on routinely processed materials, but capable of highly detailed single cell analysis.

## Materials and Methods

### Patients and case selection

21 cases of leiomyosarcoma were selected out of 77, based on both full clinical history and tissue block availability. The female patients were aged 51.2 ± 11.8 years (34–69), 52% post-menopausal, 11 FIGO stage IB, 1 stage IIB, 5 stage IIIB/C, 3 IVB, one unknown. 13 were classified TNM pT1, 1 pT2, 6 pT3. The biological parameters are reported in Table 1.

**Table 1:** Clinicopathologic and phenotypic data.

Case N.	Age	Menopause	FIGO	TMN	Size cm.	Mitosis/10HPF	Necrosis	MHC Class I	MHC Dynamics	Desmin	B7H3	Axl	IDO	Total cells/sq mm	Segmented/total DAPI	Predominant T cell phenotype
1	39	No	IVB	pT3aNxM1	6	20	Present	+++	induced	+	+ het	++	pos	900	31%	T_ex_-chronic
2	63	Yes	IVB	pT1bNxM1	16	11	Present	+++	constitutive	+	+	±	pos weak	1248	23%	T_ex_-acute
3	34	No	N/A	N/A	2	9	Absent	+	induced	+++	+ het	++	pos	517	15%	Deserted
4	36	No	IB	pT1bN0Mx	11	50	Present	±	constitutive	±	+++	++	pos	210	5%	Deserted
5	55	Yes	IIIB	pT3bNxMx	20	18	Absent	+	induced	neg	+++	++ het	pos	2567	77%	T_ex_-acute
6	61	Yes	IB	pT1bNxMx	12	25	Focal	+	constitutive	+++	+ het	+ het	pos	474	8%	Deserted
7	44	No	IVB	pT1bNxM1	14	40	Present	+	constitutive	neg		±	pos	363	6%	T_ex_-acute
8	40	No	IIIB	pT3bN0Mx	4	30	Present	±	constitutive	+++	+het	+	pos	481	8%	Deserted
9	47	No	IB	pT1bN0Mx	9	35	Focal	+++	constitutive	+	+ het	neg	pos weak	593	14%	Deserted
10	62	Yes	IB	pT1bN0Mx	8	25	Focal	+++	induced	+++	+/− het	+	pos weak	1780	38%	T_ex_-acute
11	61	Yes	IIB	pT2bNxMx	13	25	Focal	+++	constitutive	neg	+	+	pos	1644	44%	Deserted
12	49	No	IB	pT1bNxMx	11	25	Focal	+ focal	induced	neg	+++	±	neg	2890	54%	Deserted
13	59	Yes	IB	pT1bNxMx	7	13	Focal	++ het	induced	neg	+++	++	pos weak	3533	93%	T_ex_-acute
14	35	No	IB	pT1bNxMx	11	50	Absent	+ het	induced	++	+	++	pos	2756	33%	T_ex_-mixed
15	57	Yes	IIIB	pT3bN0Mx	7	20	Present	+	constitutive	++	++ het	neg	neg	72	2%	Deserted
16	49	No	IIIC	pT3bN1Mx	14	25	Present	+++	induced	neg	−	+	neg	1631	33%	T_ex_-chronic
17	60	Yes	IB	pT1bN0Mx	19	11	Focal	+	induced	neg	−	++	pos	984	50%	T_ex_-mixed
18	71	Yes	IB	pT1bN0Mx	9	35	Focal	+	constitutive	+	−	++	pos	645	13%	T_ex_-mixed
19	51	Yes	IIIC	pT3bN1Mx	19	60	Present	+++	constitutive	+	+/− het	++het	neg	1296	27%	T_ex_-chronic
20	69	Yes	IB	pT1bNxMx	10	40	Present	±	induced	++	−	++	pos het	666	21%	Deserted
21	34	No	IB	pT1bN0Mx	3	10	Absent	++ het	constitutive	+++	−	++	pos	502	10%	Deserted

NOTE: Abbreviations: FIGO: International Federation of Gynecology and Obstetrics staging system; TMN: standard for classifying the extent of spread of cancer; HPF: high power fields; het: heterogeneous; Total cells/sq mm: total number of cells in the core, based on DAPI staining; T_ex_: T cell exhausted. The predominant T cell phenotype was defined based on PD1 vs TCF7 expression: High PD1 – low/absent TCF7: acute exhaustion. Low PD1 – TCF7 present: chronic exhaustion. (See supplementary data).

The study has been approved by the Institutional Review Board Comitato Etico Brianza (https://www.asst-monza.it/comitato-etico), N. 3204, “High-dimensional single cell classification of pathology (HDSSCP)”, October 2019. Patients consent was obtained or waived according to article 89 of the EU general data protection regulation 2016/679 (GDPR) and decree N. 515, 12/19/2018 of the Italian Privacy Authority.

Two random 2 mm cores of non-necrotic, non-sclerotic, full tumor tissue for a total of 6.28 mm^2^ per case (equivalent to 40 HPF) were placed in a Tissue Microarray block (TMA model CK4500, Integrates Systems Engineering srl, Milan, Italy).

### Multiple iterative labeling by antibody neodeposition (MILAN)

Dewaxed, antigen retrieved 3 µm sections were processed for indirect IHC or multiple labeling as previously described in detail^24,25^ and Supplementary Data.

Briefly, the sections were incubated overnight with optimally diluted primary antibodies in combination of four, washed and counterstained with four distinct fluorochrome-tagged secondary antibodies^25^. The slides, counterstained with DAPI and mounted, were scanned on an S60 Hamamatsu scanner (Nikon, Italia) at 20x magnification, after which the stains were removed with a beta-mercaptoethanol and sodium dodecyl sulphate mix, extensively washed and re-staind for the subsequent markers^25^. Re-staining a sample of antigens after about 30 cycles showed no consistent antigen loss and occasional increased antigenicity (Supplementary Fig. 1). The list of primary and secondary antibodies is available in the Supplementary Materials.

### Preparation of immunofluorescent images for image analysis

Single.ndpi images for each case were registered via alignment of DAPI nuclear stained images with Fiji, saved as.tiff files and autofluorescence was subtracted^25^.

Two single cell masks were produced for each case with Cell Profiler (2.2.0)^26^ by segmentation of thresholded images: a DAPI mask encompassing all the nuclei and a mask obtained by the combined CD43, CD45, CD68 and CD163, henceforth named “targeted mask”. Regions of interest composed of small portions of dendritic-shaped cytoplasm were excluded digitally (see Supplementary Methods).

Comparison of the total cell yield and detailed high-dimensional phenotypic content obtained with the DAPI vs the targeted masks showed variable but constantly inferior cell number representation with the former (36%, 65%, 95%of the targeted mask-derived cells) and loss of minor phenotypic subsets by tSNE and Phenograph clustering (not shown). This because macrophages have reduced chromatin DAPI staining and do not provide enough contrast to be detected by threshold algorithms.

### High-dimensional analysis

Individual masks and .tiff files from all cases were loaded in HistoCAT^27^, data obtained from the image analysis were processed by dimensional reduction and unsupervised clusterization algorithms, t-SNE and Phenograph plots were generated. Image analysis data were subsequently exported as .csv files. In order to analyze the infiltrate composition of individual cases, we developed an R script (Supplementary Methods) to systematically process all cells of all cases identified by image analysis. Phenoclusters were plotted as heatmaps (Supplementary Fig. 2) with a custom R script, to allow the identification of cell composition. Each heatmap was inspected for specific lineage- or function-associated markers, with the requirement that each defining marker needed to be expressed at high levels (clearly visualizable by a blue-red divergent color palette) in a third or more of the cells. A nine cell-type classification of inflammatory infiltrate was obtained by the inspection of all generated heatmaps and was based on criteria listed in Supplementary Table 1. The cell content of each phenogroup was used as the numerator to quantify the percentage of a given marker or phenotype.

### Neighborhood analysis

An unbiased quantitative analysis of cell-cell interactions was performed using an adaptation of the algorithm described in^27^ for neighborhood analysis to systematically identify social networks of cells and to better understand the tissue microenvironment (Supplementary Methods).

### PTEN status by FISH

*In situ* hybridization for PTEN was performed with the ZytoLight SPEC PTEN/CEN 10 dual color probe (ZytoVision GmbH, Germany) for the centromeric and the gene-specific regions of chromosome 10.

## Results

The clinicopathologic data of the 21 sarcomas are reported in Table 1.

### The inflammatory infiltrate

**Figure 1:**
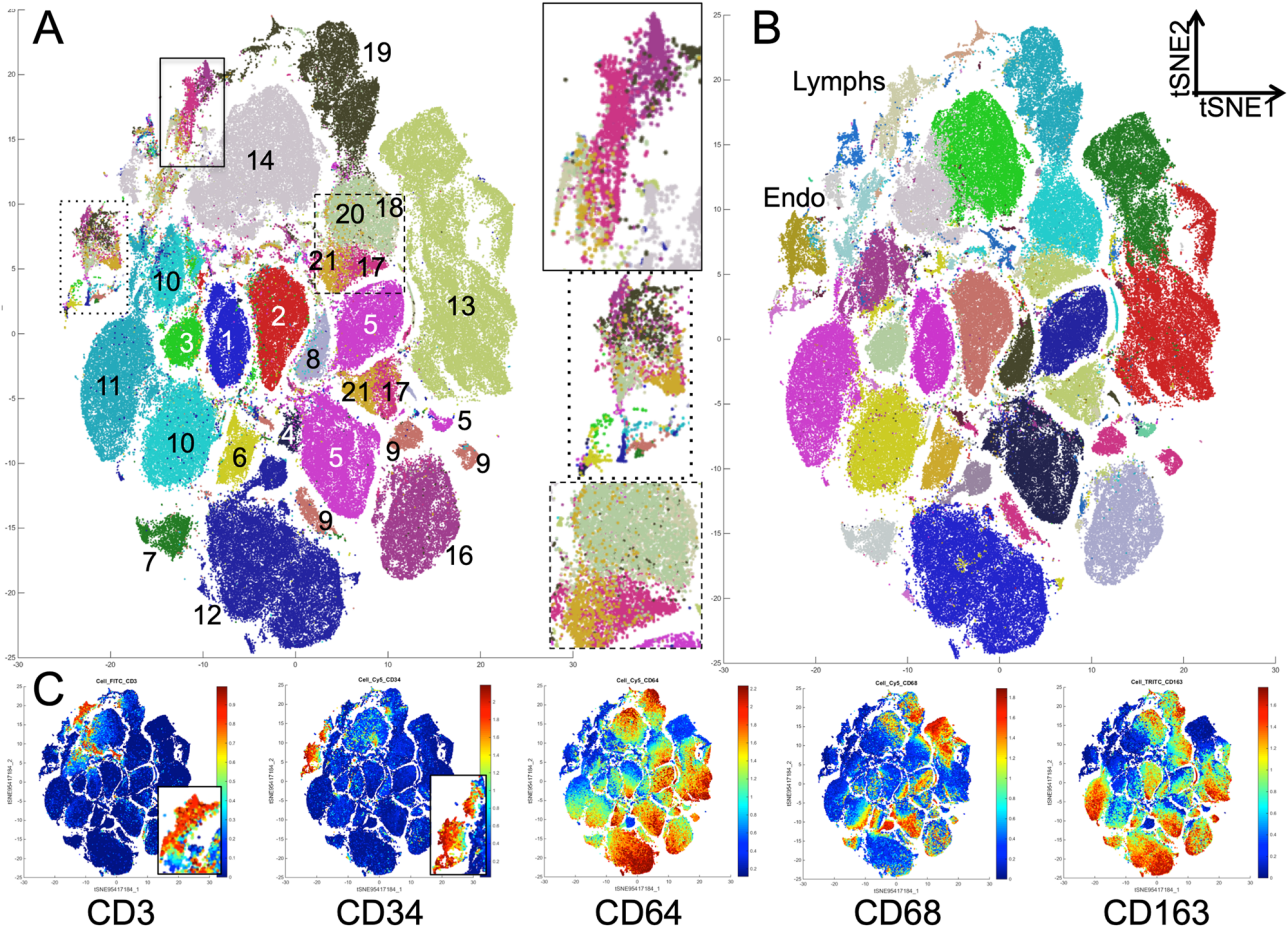
The lymphocyte and endothelial phenotypes are shared among the sarcoma cases but each one has an individual macrophage population. (**A**) tSNE plot of all 21 cases. Each case is color-coded and marked by the case number. On the right are enlarged portions highlighted on the plot. Note admixture of the cases in the boxed areas and in cases 17, 18, 20 and 21. Case 15, containing very few cells, is not marked. (**B**) Phenograph groups are plotted on the tSNE plot shown in A. Note the lymphocytes and the endothelial phenogroups, corresponding to the areas of case admixture shown in A. Macrophage populations for each case is represented by one to three phenogroups. (**C**) tSNE plots are highlighted with lymphoid (CD3; enlarged in the inset), endothelial (CD34; enlarged in the inset) and myelomonocytic markers.

High-dimensional analysis of all 21 cases showed a majority of independent, non-overlapping clusters of myeloid phenotype, one or two per case, and smaller overlapping clusters, comprising T-cells and endothelial cells (Fig. 1). Only in four instances (cases N. 17,18, 20, 21) myeloid phenoclusters from separate cases did overlap (Fig. 1).

**Figure 2:**
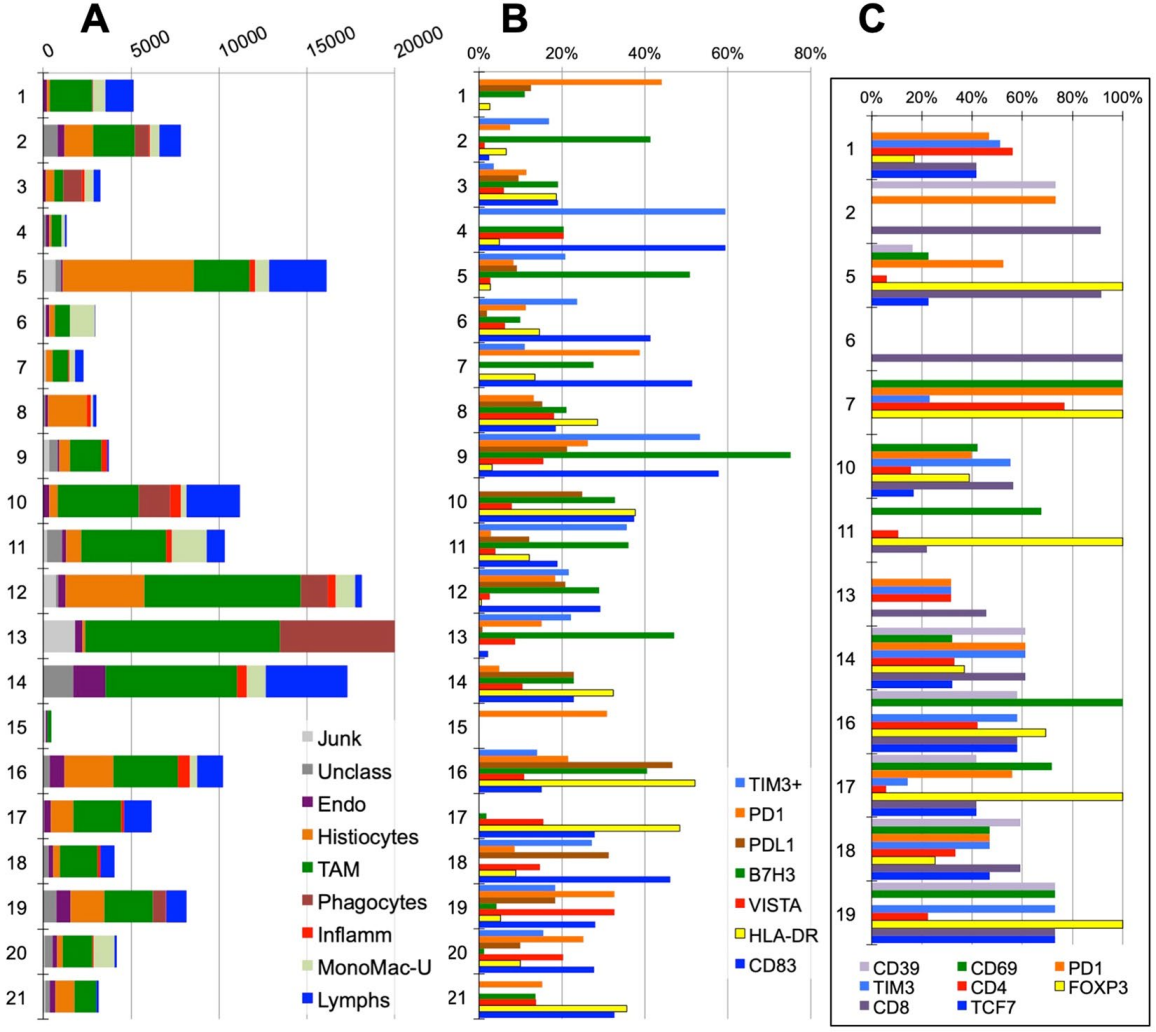
Composite phenotype of the myelomonocytic and lymphoid infiltrate. (**A**) Absolute numbers of the inflammatory cells in each case per 6.28 mm^2^. Note the selective absence of CD16+TAMs in case 8, non neoplastic myometrium. Legend is shown in the bottom right of the graph. (**B**) Distribution of checkpoint protein and activation markers on myelomonocytic cells. Case 8, non neoplastic myometrium, has a small percentage of inflammatory cells with a coordinated activated phenotype; in all other cases, the expression of markers is uncoordinated. Legend is shown in the bottom right of the graph. (**C)** Distribution of relevant markers on lymphoid subsets. Note that only cases with enough lymphocytes are represented. CD39, CD69, PD1 and TIM3 are expressed as percentage of all CD3+ lymphocytes. FOXP3 percentages refer to the CD4+ subset. TCF7 refers to the CD8+ subset. Legend is shown at the bottom of the graph.

Thus, the majority of the infiltrating inflammatory cells in each case is composed of macrophages whose phenotype reflects the unique biology of each tumor (Fig. 2), and a minor population of T-cells.

In order to understand the composition of the inflammatory infiltrate, each sarcoma case was analyzed separately in high-dimension (Supplementary Figs. 2 and 3).

### Lymphoid cells

TILs, almost exclusively T-cells and NK-cells, represents 3%-29% of the inflammatory infiltrate (0.3%-15.3% of the total sample cellularity), the rest being myelomonocytic cells (Fig. 2, Supplementary Table 2 and Supplementary Data). A few B cells in one case and no plasma cells were identified.

TILS were composed of 30% ± 22% CD4+, 62% ± 23% CD8+ and 9% ± 8% NK-cells.

CD4+ T-cells were 68% ± 36% FOXP3+, largely negative for activation markers (OX40, CD69,CD32). (Fig. 2, Supplementary Table 2 and Supplementary Data).

**Figure 3:**
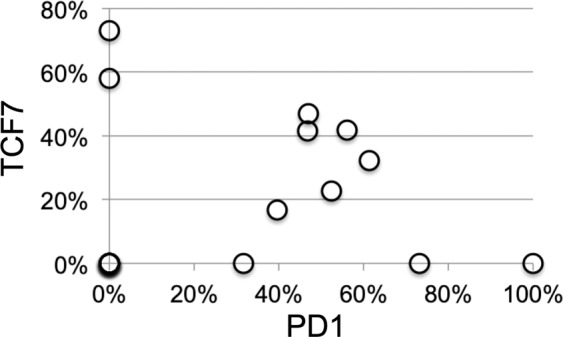
Relationship between PD1+ and TCF7+ CD8+ T cells subsets. The coexistence of PD1+ TCF7− and of TCF7+ PD1− CD8+ T cells in each case is plotted as percentage of all CD8+ cells. Note that some samples show skewed expression by either population, others have a mixture of both. For complete data see Supplemental Data.

**Figure 4:**
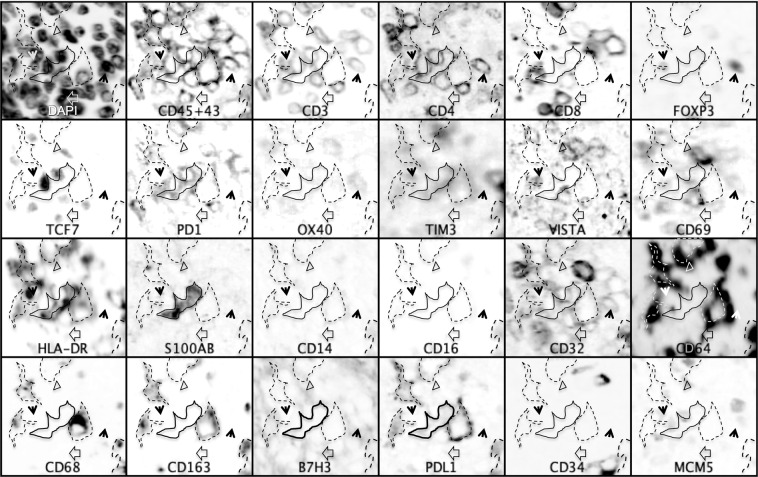
T lymphocyte, macrophage and dendritic cell interactions in sarcomas. Inverted grayscale images of immunostained section detail from Case 14. PDL1+ macrophages expressing HLA-DR, CD16, CD64, CD68, CD163 and TIM3 are highlighted by a dashed line and are surrounded by PD1+ T cells, both CD8 and CD4. In the center, an HLA-DR+, S100AB+ CD14- dendritic cell is highlighted by a solid line. The empty arrowhead shows a CD8+, VISTA +, TCF7+ lymphocyte. The black arrowheads point to CD4+, FOXP3+, TCF7−, CD32±, OX40 ± regulatory T cells. The empty triangle points to a CD3+, CD8+, TCF7+, CD69+, VISTA+ lymphocyte. Note that TCF7 and FOXP3 are mutually exclusive. Negative markers are not represented. Image size 60 × 56 µm.

CD8+ T-cells were identified as distinct phenoclusters in about half of the cases, whenever a sufficient number of TILS was present. In those cases, often multiple phenotypically distinct phenoclusters were detected per case, displaying evidence of activation (CD69) and exhaustion (PD1, TIM3, VISTA, CD39). VISTA+ T-cells were observed in 8 cases, largely CD8+ TCF7−. TCF7, a transcription factor linked to resident memory phenotype and reactivation, was contained in 42% ± 18% of CD8+ cells, in an inverse relationship with PD1 (Figs. 3 and 4).

Lymphocytes expressing GranzymeB and Granulysin, partly CD8+, were identified as a separate phenocluster in some cases or as part of a single cluster of cells in samples with fewer TILs.

NK cells, defined as CD45+ CD3− and expressing GranzymeB and Granulysin, were a minority of lymphoid cells in abundant infiltrates only.

### Myelomonocytic cells

**Figure 5:**
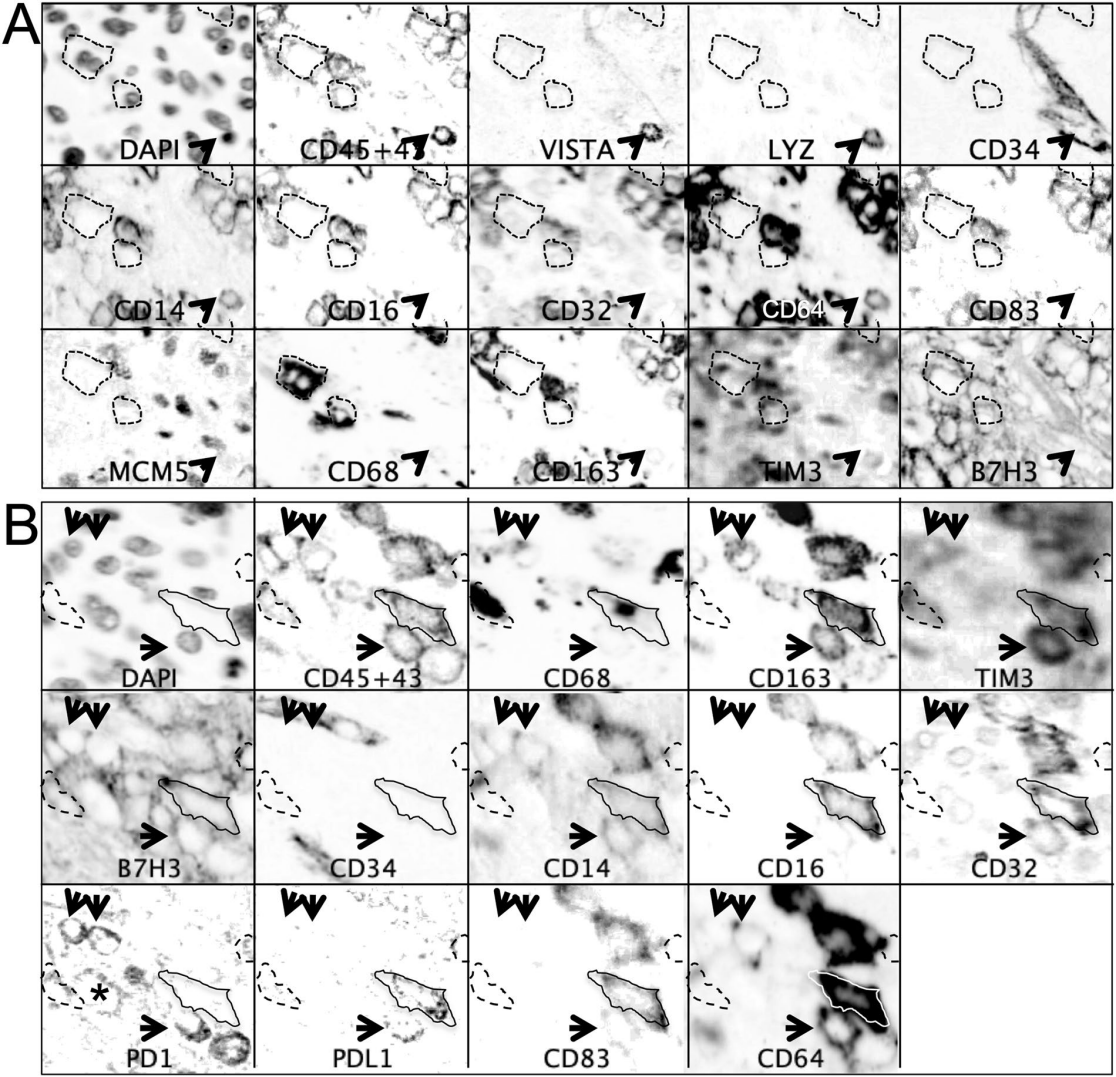
Heterogeneity of macrophages in sarcomas. Inverted grayscale images of immunostained section detail from Case 13. (**A**) CD68+, CD14−, CD16−, CD64− CD163 phagocytes are highlighted by a dashed line. The arrowhead points to an endovascular VISTA+, LYZ+, CD14+ inflammatory monocyte. Negative markers are not represented. Image size 105 × 145 µm. (**B**) A solid line highlights an activated CD83+ PDL1+ TAM (CD14+, CD16+, CD32+, CD64+, CD68+, CD163+, TIM3+). The arrows point to PD1+ Monocytes/Macrophages undefined (CD14+, CD64±, CD68±, CD163±), one of which co-express PD1 and PDL1. The dashed outlines indicate PD1+, CD68+, TIM3± Phagocytes. Note a PD1+ isolated tumor cell (asterisk). Negative markers are not represented. Image size 76 × 67 µm.

Myelomonocytic cells represents 64% ± 13% of the infiltrate (Fig. 2, Supplementary Table 2 and Supplementary Data) and contained the most diversified cell type, often represented by multiple distinct phenogroups within each case (Fig. 5).

The most distinctive group, named Tumor Associated Macrophages (TAM; 38% ± 13%) expressed CD16 and restricted lineage markers (CD68, CD163) and was consistently present in all tumor cases, but only occasionally in the non-neoplastic samples (Case #8 and 3 normal myometria, not shown). TIM3 expression among myelomonocytic cells was restricted to this cell type and to the inflammatory monocytes (see below).

The sarcomas contained CD16-negative histiocytes (20% ± 17%) and phagocytes (CD68+ CD163−; 5% ± 10%), these latter in 7/21 cases.

Phagocytes and Undefined Monocyte-Macrophagic cells (9% ± 12%) were characterized by the absence or spotty presence of several monocytic lineage markers (CD14, CD64, lysozyme/LYZ, CD163), PD1, PDL1, VISTA, CD83, B7H3.

All cases but five contained a small but very distinct population of small monocytic cells, often intravascular and proliferating, with a distinctive LYZ+ VISTA+ phenotype, which we defined “inflammatory” because of the association with the inflammatory infiltrate. These cells were often CD14+, CD16^low^ or negative, sometimes TIM3+, but devoid of other myelomonocytic markers.

### Spatial relationships between lymphoid and myelomonocytic cells

A neighborhood analysis between all phenotypic subsets revealed a substantial mutual avoidance across the phenotypes and clustering together of similar cell types, both at submicroscopic (<100 µm range) and at microscopic range (>100 µm range) (Supplementary Fig. 4). TILs avoid close contact with checkpoint-bearing macrophages (Supplementary Fig. 4 and Methods).

### Tumor cells

Tumor cells had histocompatibility antigens staining patterns which could be defined as constitutive and induced; in the first case tumor cells were all positive or negative throughout the sections (Supplementary Fig. 5). In the second case, tumor cells admixed with the inflammatory infiltrate showed increased staining, compared to non-inflamed portions (Supplementary Fig. 5).

No HLA-DR was observed on tumor cells.

Tumor cells did not expressed most of the markers tested, including PDL1, with the notable exception of MCM5, IDO, B7H3 and Axl (see Table 1 and Supplementary Fig. 5). Occasional PD1+ tumor cells were observed in some cases (Fig. 5B and Supplementary Data).

CD34+ endothelial cells stained for VISTA and B7H3 in about half of the cases, often in a subset^28^ (Supplementary Fig. 5).

The vast majority of the cases tested were either diploid or had loss of one copy of the PTEN gene in 3.1%-22.1% of the cells (Table 1), with only one case (case #7) carrying homozygous PTEN deletion in 13.2% of the cells.

## Discussion

Data about the inflammatory infiltrate in untreated primary sarcomas are scarce. We detail here the composition of the innate and adaptive arm of the response of the host to a soft tissue tumor.

We found a variety of individual phenotypic profiles, not unlike other reports^29,30^.

In 11/21 cases we found a T-cell phenotype consistent with antigen exposure and acute or chronic stimulation, leading to exhaustion, mostly in tumors HLA Class I+.

The remaining 10 cases that we classified as “deserted” had usually less than 60 lymphocytes per mm^2^ and 0.3%-4.2% of total cellularity, with a phenotype suggestive of passer-by. These tumors were all negative or weakly expressing HLA-A,B,C, with two exceptions.

Each of the cases hosting antigen-experienced T-cell phenotypes displayed an unique combination of cell markers, which could be summarized as acutely exhausted CD8+ T cells, chronically exhausted CD8+ and FOXP3+ CD4+ T cells. These phenotypes were reminiscent of T-cells acutely exposed to a persistent antigen such as a virus or a neoantigen, bearing PD1, CD69, CD39^31,32^ and TIM3^33^, and induced to anergy (exhaustion)^3^. Others expressed TIM3, lower levels of PD1 and TCF7 to a variable amount (17–73%), resembling exhausted CD8+ T-cells chronically exposed to an antigen, but in a resident memory state, susceptible of reactivation upon re-stimulation or therapeutic checkpoint inhibition reversal^33,34^. A mixture of these two cell types was sometimes present. It has been reported that up to a third of the cases displaying a CD8+ TCF7+ phenotype may benefit from a checkpoint blockade therapy^33^.

CD4+ T-cells had a FOXP3+ regulatory phenotype, occasionally as the minor population, as shown in other cancer models^35^.

The variability of T-cell phenotypes found between and within cases is suggestive of an ongoing editing of the adaptive immune response^36^, on a case-by-case basis.

The tumors we have examined are full blown malignant tumors of low mutation rate, yet, as shown in a single successful immunotherapy case^22^, this may induce an anti-tumor T-cell response. ULMs are also conspicuously devoid of HLA-DR+ CD14− dendritic cells and tertiary lymphoid structures, thus an adaptive response may occur in tumor-draining lymph nodes.

The failure of single-agent checkpoint inhibitor therapy in several published cases^21–23^ is at odd with our findings. One hypothesis is that the antigen to which the adaptive immune system responds is not a tumor antigen^37^. In the single ULM case studied^22^, T-cells were tumor antigen-specific and responded. None of our cases except one (Table 1) had homozygous deletion of PTEN, associated with an immunosuppressive tumor phenotype^22^.

Alternatively, failure to elicit an anti-tumor response may be caused by an independent immunosuppressive effect brought by the inflammatory infiltrate, chiefly the infiltrating macrophages.

Macrophages, and in particular tumor-associated macrophages (TAMs) have been shown to mediate the suppression of an anti tumor response^38,39^. A dichotomic view of alternatively polarized macrophages has let to a more nuanced picture^40^, where there is a dynamic equilibrium between various defined stages of macrophage polarization.

As published by others^16,41^, we found a range of leukocytes (CD45+, CD43+) with myelomonocytic differentiation. Differently from lymphocytes, whose aggregate phenotype was conserved across all cases, myelomonocytic cells had unique, case-specific populations. A tissue-restricted secretome influencing macrophages has been described^19^; a similar effect may occur via monoclonal sarcoma cells, yielding a highly diversified, stimulus-driven differentiation reflecting individual tumor-specific microenvironment^30,42,43^. As shown by others^30,38,44^, the tumor harbors multiple subsets, often not found in the normal counterpart neither described before.

Furthermore, our neighborhood analysis of the macrophage subsets show both a submicroscopic and a microscopic local differentiation, producing a checkered pattern, which has to be kept in mind in order to sample the tissue adequately, as we did with larger (2 mm), multiple TMA cores.

Despite the large variety of individual phenotypes, by analyzing single cases with multiple markers we could identify five rather consistent groups present in all cases: histiocytes, phagocytes, TAM, inflammatory monocytes and Monocytes/Macrophages, undefined.

With the exception of TIM3, largely restricted to CD16+ TAMs, all the immuno-modulating markers were expressed in a minority of cells and were distributed across the five subsets.

We observed PD1, previously reported in circulating monocytes in HIV and in M2-type TAMs^45^ expressed on macrophages in 18/21 cases, occasionally with activation markers such as HLA-DR, OX40 and/or CD83. Interestingly, non-lymphoid PD1 expression in FFPE material could only be detected with UMAB197; this antibody has a broader reactivity than other PD1 antibodies and detects the molecule on B cells, monocytes and tumor cells, as reported by others^45,46^. Subtle subcellular variations in membrane staining by each antibody we tested (Supplementary Fig. 6) may has to do with the recognition of glycosylation-dependent, thus cell type restricted epitopes on PD1.

Consistent with the mouse data^45^, PD1 was expressed on 27 phenogroups, only two of them HLA-DR+ and CD83+.

Markers of activation (HLA-DR, CD83, OX40) were found on macrophages in almost all cases in all subsets, except on the inflammatory monocytes. Often activation was matched with proliferation (MCM5+).

More complex to understand was the expression of members of the immunological synapsis (PD1, PDL1, B7H3, VISTA), which, with the exception of TIM3, restricted to TAMs, were displayed by several subsets.

PDL1, expressed by myelomonocytic cells in 15/21 cases, is a ligand for PD1 and CD80 on cognate T-lymphocytes^6,7^ and a constitutively negative signaling molecules on macrophages^47^. Engagement of PDL1 induces proliferation, survival and upregulation of MHC Class-II, CD86 and cytokine secretion, promoting a proinflammatory phenotype^47^. In our PDL1-negative sarcoma cases, there is little relationship between activation and PDL1 expression in macrophages; of the 24 PDL1+ phenogroups, only seven express HLA-DR and eight CD83. These data hint at a lack of engagement of this molecule on macrophages.

VISTA-expressing macrophages have been described in prostate cancer^48^, particularly after chemotherapy; we do see this phenotype largely restricted to endocapillary LYZ+ VISTA+ monocytes, occasionally proliferating, which could be the seeding population from the bloodstream.

Human macrophages found in tumors may be able to present antigens to T-cells^41^, where the co-inhibitory molecules act to protect the macrophage from cytotoxicity during the encounter. We failed to demonstrate a statistically significative close interaction between T-cell subsets and checkpoint molecules-bearing macrophages, except for the suggestive images provided by tissue snapshots (Fig. 4). This may has to do with the highly motile properties of T-cells, whereby during an asynchronous activation of multiple immune synapsis, the challenged T-cells may distance themselves from the interactor by the time the phenotype has changed to reflect the effect of the challenge^49^.

In summary, we have described a variety of innate and adaptive immune cell phenotypes in ULMs, suggestive of antigen experience, and exploitable for a targeted immune intervention, despite the variability in phenotypes, particularly on the macrophage side.

## Supplementary information

Supplementary information.

SupplementaryData-Manzoni.xlsx.
