# Author Correction: The Adaptive and Innate Immune Cell Landscape of Uterine Leiomyosarcomas

**DOI:** 10.1038/s41598-021-95855-1

**Published:** 2021-08-05

**Authors:** Marco Manzoni, Maddalena M. Bolognesi, Asier Antoranz, Rosanna Mancari, Silvestro Carinelli, Mario Faretta, Francesca M. Bosisio, Giorgio Cattoretti

**Affiliations:** 1 Pathology, Department of Medicine and Surgery, Università di Milano-Bicocca, Via Cadore 48, Monza, (MI), Italy; 2 National Technical University of Athens, Zografou Campus, 9 Iroon Polytechniou str., 15780 Zografou, Athens, Greece; 3 Division of Gynecologic Oncology and Pathology, European Institute of Oncology, Via Ripamonti 435, 20141 Milan, Italy; 4 Department of Experimental Oncology, European Institute of Oncology, IRCCS, via Adamello 16, 20139 Milan, Italy; 5 Laboratory of Translational Cell and Tissue Research, KU Leuven, Herestraat 49, 3000 Leuven, Belgium

Correction to: *Scientific Reports* 10.1038/s41598-020-57627-1, published online 20 January 2020

The original version of this Article contained an error in Affiliation 4, which incorrectly given as ‘Department of Experimental Oncology, European Institute of Oncology, via Adamello 16, 20139, Milan, Italy.’ The correct affiliation is listed below:

Department of Experimental Oncology, European Institute of Oncology, IRCCS, via Adamello 16, 20139, Milan, Italy.

The original Article has been corrected.

