# Supplementary material for: The Adaptive and Innate Immune Cell Landscape of Uterine Leiomyosarcomas
Source: Sci Rep. 2020 Jan 20;10:702. doi: 10.1038/s41598-020-57627-1 (PMC6971074; doi:10.1038/s41598-020-57627-1)
Supplement: Supplementary file 1 — Supplementary information. [file 41598_2020_57627_MOESM1_ESM.pdf]

THE ADAPTIVE AND INNATE IMMUNE CELL LANDSCAPE OF UTERINE LEIOMYOSARCOMAS

Marco Manzoni, Maddalena M Bolognesi, Asier Antoranz, Rosanna Mancari, Silvestro Carinelli, Mario Faretta, Francesca M Bosisio, Giorgio Cattoretti

SUPPLEMENTARY METHODS

Conventional immunohistochemistry.
Conventional, horseradish peroxidase (HRP) and DAB-based immunohistochemistry (IHC) was performed a previously published [1] for HLA Class I and CD45 antigens. No other MHC-associated molecules were tested, except the ones listed.

Multiplex indirect immunofluorescence.
Dewaxed, antigen retrieved 3 µm sections were processed for multiple labeling as previously described in detail in the MILAN method [2,3]. Four antibodies per run were selected with one of these two combinations: a) a rabbit and three mouse antibodies of different isotype (e.g. one IgG1, one IgG2a, one IgG2b etc.); b) antibodies raised in four different species: mouse, rabbit, rat and goat. In a) fluorochrome conjugated, isotype-specific, absorbed goat antibodies were employed. In b) donkey species-specific secondary antibodies were used. The concentration of the primary Ab was kept at 1µg/ml or equivalent by dilution and incubated overnight. Secondary antibodies were used at 5µg/ml and in the case of BV480 conjugates, 7.5 µg/ml. All incubation times besides the first were 30' at room temperature, with two washes in Tris-buffered saline pH 7.5 to which 0.1 M sucrose and Tween-20 were added (see [2]). Enhancement of the staining was accomplished by a second incubation with isotype/species and concentration matched irrelevant immunoglobulins, followed by washing and the application of the same secondary antibody combination.
Sections were counterstained with DAPI 5 µM and mounted in Glycerol-TBS-sucrose with 0.2% n-propylgallate.
For scanning, sections were placed in an Hamamatsu S60 scanner (Nikon, Italy) and scanned with the NDPI scanner software. Slide were focused in brightfield and the whole section acquired for each of the four fluorescent channels, DAPI and autofluorescence [3].
The coverslip was removed by immersing the slides in TBS and allowing enough time for the coverslip to float.

Validation of extended staining and stripping cycles.
While the current experiment entails only 10 staining and stripping rounds (see Supplemental Data Excel file), an independent experiment was run, comprising 27 successive rounds of staining and stripping, in order to validate the robustness of the method. The antibody stainings included in the first five runs were repeated after a five months interval by running the very same tonsil sample with the very same dilutions of the primary and secondary antibodies, kept at +4C in vertical mailers [2,3]. Second time acquisition was performed with the very same

exposure time as the first for each channel. CD4, CD83 and BCL6 repetition was after round 28, HLA-DR and CD1c after round 29, IRF4, PDL1 and PRDM1 after round 30. Pre- and post-repetition images for each antibody was aligned as described [3], autofluorescence subtracted and the mean intensity of representative areas compared as published [4]. Pixel-by-pixel comparison of early and late stainings for the same antibody showed no cell loss and no new pixels revealed for the two most enhanced staining, CD4 and PDL1 (Supplemental Fig. 1).

Generation of registered gray level images for analysis.
Single .ndpi images for each case were registered via alignment of DAPI nuclear stained images with Fiji, saved as .tiff files and autofluorescence (AF) was subtracted [3].

Cell masks generation.
From default thresholded, segmented DAPI images of single cases a mask was created (DAPI mask) with Cell Profiler (2.2.0) [5] in order to count the total number of cells per case. A selected multimarker approach, based on a single mask composed by the sum of optimally contrasted, AF-subtracted images of CD43, CD45, CD68 and CD163, henceforth named "targeted mask", was generated and used to analyse the inflammatory infiltrates. In order to control for the creation of regions of interest (ROIs) composed exclusively of small portions of dendritic-shaped cytoplasm, incorrectly labeled as individual cells, the size for segmentation in Cell Profiler (and in ImageJ) was set at 15-150 pixel of diameter. Comparison of the total cell yield and detailed high-dimensional phenotypic content obtained with the DAPI and the targeted masks showed variable but constantly inferior cell number representation with the former (36%, 65%, 95%of the targeted mask-derived cells) and inadequate identification of minor phenotypic subsets by tSNE and Phenograph clustering (not shown). This because macrophages and dendritic cells have reduced chromatin staining with DAPI and do not provide enough contrast to be detected by threshold algorithms in the presence of other kind of cells.

High-dimensional analysis by Histocat and R script
Individual masks and .tiff files from all cases were loaded in HistoCAT [6], data obtained from image analysis were processed by dimensional reduction and unsupervised clusterization algorithms, t-SNE and Phenograph plots were generated. Image analysis data were subsequently exported as .csv files. In order to analyze the infiltrate composition of individual cases, we developed an R script to systematically process all cells of all cases identified by image analysis, with the use of t-SNE and PhenoGraph algorithms. Cellular elements whose area exceed the 175 μm^2 - 700 μm^2 area range were excluded from the analysis. Biomarkers intensity values were stored in a dataframes list, which was further transformed in a list of numerical normalized matrices. In order to identify the specific cell population of each cluster we employed a fully automatic plotting of heatmap of each clusters which reordered both rows (cells) and columns (markers) in a hierarchical clustering fashion by the use of the Ward.D2 algorithm (Supplemental Fig.2). This allowed the identification of the cell composition and uniformity of each clusters. Each heatmap was linked to an Excel file list for annotation and inspected for specific lineage- or function-associated markers, with the requirement that each defining marker needed to be expressed at high levels (clearly visualizable by a blue-red divergent color palette) in a third or more of the cells. Each of the 303 phenoclusters was

classified with a nine cell-type classification of inflammatory infiltrate, based on criteria listed in Table 2. After assigning a cellular classification to each cluster, the phenogroups were redrawn (Supplemental Fig.3). The cell content of each phenogroup was used as the numerator to quantify the percentage of a given marker or phenotype.

Neighborhood analysis

An unbiased quantitative analysis of cell-cell interactions was performed using an adaptation of the algorithm described in [6] for neighborhood analysis to systematically identify social networks of cells and to better understand the tissue microenvironment. Our adaptation also uses a kernel-based approach (radius = 6.67 px = 3 µm) to define the neighborhood of a cell and a permutation test (N = 1000) to compare the number of neighboring cells of each phenotype in a given image to the randomized case. This allows the assignment of a significance value to a cell-cell interaction in each case representative of the spatial organization of its cells. Significance values were further classified into: avoidance (-1), non-significant (0), and proximity (1) using a significance threshold of 0.001 (more significant that all the random cases). Interactions across images were integrated according to equation 1:

$$P_{i,j} = \frac{\sum_{k=1}^{M}\left(c_{i,j,k} \cdot \sqrt{N_{i,j,k}}\right)}{\sum_{k=1}^{M}\left(\sqrt{N_{i,j,k}}\right)}$$

where $c\_(i,j,k)$ is the significance value (-1, 0, or 1) of the interaction between cell types *i* and *j* for image *k*, and $N\_(i,j,k)$ is the geometric average of the number of cells of type *i* and *j* for image *k*. Cell-cell interactions were considered strong if they were significant in at least 75% of the N-adjusted cases (abs(P) > 0.75), moderate if 50%, (0.5 < abs(P) <= 0.75), weak if 25% (0.25 < abs(P) <= 0.5), and non-significant otherwise (abs(P) <= 0.25).
An interaction was considered when the edges of the modelled cells are <3 µm from each other with a resolution of 0.45 µm per pixel.
In order to interrogate each sample for close interactions (<3 µm) of significance between subsets of CD8 or CD4 lymphoid cells and checkpoint molecules-bearing macrophage subsets, all phenogroups containing macrophages were reassigned to a PDL1+ group, to a group expressing all checkpoint molecules (PD1, TIM3, B7H3, VISTA but PDL1), and to a group devoid of such molecules. Unclassified and inflammatory monocytes were excluded from the analysis, as all other non-lymphoid, non-macrophagic subsets.

PD1 staining validation.

Four antibodies against PD1 did stained FFPE sections: two mouse IgG2a (UMAB197 and UMAB199), one mouse IgG1 (NAT105) and a goat polyclonal. This latter has been raised against the N terminal AA 25-167. The target sequence of the others is either proprietary or unknown. UMAB199 uniquely recognizes PD1 on peptide arrays, while UMAB197 also recognizes a TRMT112 peptide on peptide arrays, but not on Western Blots (Origene, personal communication). In tissues the staining pattern of UMAB197 does not corresponds to TRMT112, as shown by two non-overlapping antibodies of the Human Protein Atlas

(https://www.proteinatlas.org/ENSG00000173113-TRMT112/tissue/primary+data). By aligning PD1 (Uniprot Q15116) and TRMT112 (Q9UI30) in Clustal Omega (https://www.ebi.ac.uk/Tools/msa/clustalo/), a discontinuous stretch of homology is detected toward the C-terminus of PD1 at peptides 221-224 and 233-236. Because of the reactivity with immobilized peptides and on routinely processed tissues [7], we assume that UMAB197 recognizes a linear epitope, thus it must recognize the C-terminus of PD1, around AA 220-240. Both UMAB197 and 199 do detect a minor ~56 KDa molecular species, corresponding to heavily glycosylated PD1, in various human and non-human cancer cell lines of epithelial and stromal origin, in addition to a heavy band in T cell lines. This is consistent with the fact that PD1 has been detected in tumor progenitor cells in various human fresh tumors (melanoma, lung, etc.) [8,9].

To investigate the tissue distribution and co-localization of the anti-PD1 antibodies, human tonsils and the sarcomas were co-stained with either UMAB197 and NAT105 or UMAB197, UMAB199 and the goat antiserum. Sections stained with the former combination were scanned at 40x in Zeta-stack mode, acquiring 5 planar fields between -1 and +1 µm of thickness per wavelength. Subsequently, a single focus field was produced by merging all the focussed pixels with the NDPI toolkit. Detail grayscale images were inverted, and a 3D plot of the pixel intensity was obtained with FIJI (Supplemental Fig. 6).

SUPPLEMENTARY TABLES

Supplementary Table 1 Nine cell type classification of the inflammatory infiltrate.
NOTE: Myelomonocytic lineage restricted markers: CD14, CD64, CD68, CD163, LYZ. Promiscuous markers: CD4, CD16, CD32, S100AB, TIM3. Despite the fact that sparse CD16+ CD68+ macrophages are detected in 3 normal myometria, the abundance and consistency of this phenotype in leiomyosarcomas suggest to retain the term TAM for this subset of macrophages [10]. Endothelial cells, characterized by a prominent CD34 signal, were coopted by proximity in the CD43+45 mask because of encroaching lymphomonocytic cells.

SUPPLEMENTARY TABLE 2
Composition and phenotype of the inflammatory infiltrate.
NOTES: * in some samples, CD8 and KN cells are clustered in a single cluster

SUPPLEMENTARY FIGURES

Supplementary Fig. 1 Effect of repeated rounds of staining and stripping on tissue immunogenicity.
A: Comparison of tonsil immunostaining in the first five rounds (pre) of a 27+ staining sequence and the same rounds performed after 27 cycles (post). CD4, CD83 and BCL6 repetition is after round 28, HLA-DR and CD1c after round 29, IRF4, PDL1 and PRDM1 after round 30. The antibody is shown at the left. **B**: pixel-by-pixel comparison of intensity before (x axis) and after (y axis) the staining cycles; clockwise from top left: DAPI, CD4, PDL1 on epithelia and on macrophages. No cell loss is shown by the DAPI comparison. Note that in the PDL1 images, AF

was not subtracted and is represented by the horizontal species, not present after several cycles, as published before. **C**: The immunofluorescence quantification is represented as the channel variation from the pre to the post status, normalized for 256 channels. Scale bar : 100 µm. For details see supplementary methods.

Supplementary Fig. 2 Heatmap representation of inflammatory cell subsets.
Heatmaps of selected phenoclusters, representative of the inflammatory cell subsets, as defined in Supplemental Table 1, are shown. The # indicated the case, the p indicate the phenocluster number of that case. Columns are the markers, rows are the cells composing that heatmap. Each heatmap has on the left the cell classification and at the bottom right the rightmost positive markers (red), enlarged. Negative values are blue, positive red, white intermediate.

Supplementary Fig. 3 Classification of Phenogroups in Phenotypes.
Phenograph rendering of tSNE plots are shown in the top row for representative tumor samples (case N. on top). Below, the plots are redrawn with discrete phenotypic groups in color. In each column T cell (CD3) and Macrophage markers distribution on tSNE is represented. Case 8 represents non-tumoral myometrium.

Supplementary Figure 4. Neighborhood analysis of the inflammatory infiltrate.
A social network of all the components of the inflammatory infiltrate.
B strength of the interactions of all the components of the inflammatory infiltrate. Note the strength of the homotypic interactions and the degree of avoidance for heterotypic interactions.
C five examples of the spatial distribution of two subsets with strong avoidance; each figure represents two TMA cores belonging to a case, which number is indicated on top of each image.
D social network of the relationship between T cell subsets and checkpoint molecules-bearing macrophages.
E strength of the interactions between T cell subsets and checkpoint molecules-bearing macrophages.

Supplementary Fig. 5. Phenotype of tumor and endothelial cells.
A HLA-ABC staining of representative cases. In case #4 only endothelia and scattered stromal cells are positive. Case #14 shows induced HLA expression in tumor cells close to inflammatory cells (lower half). Case #11 show strong constitutive expression in all tumor cells. IHC (DAB) in light microscopy. Note that Class I is reported absent from normal myometrium [11]. Scale bar: 100 µm.
B IDO expression on sarcoma and TAMs. Case #19. Tumor cells express IDO on part of the sarcoma cells and on TAMs. In the inset a group of CD16+ TAMs is strongly IDO+. All subtypes of macrophages express IDO (not shown). Inverted IF stain. Magnification 7x.

C AXL expression in sarcomas. AXL is expressed on vasculature-associated smooth muscle cells in both cases. In case #11 the tumor cells are diffusely membrane-positive (inset). In case #5 the sarcoma is negative; only smooth muscle cells and scattered histiocytes (inset) are positive. Inverted IF stain. Scale bar: 100 µm.
D Coexpression of checkpoint molecules on endothelial cells. Case #13 shows coexpression of B7H3 and CD34. Case #12 coexpress all three. Inverted IF stain. Magnification 7x.

Supplementary Fig. 6. Identification of PD1 in tissue by antibody staining.
A comparison of PD1 UMAB197 and NAT105 staining on tonsil Germinal Center. CD3 and DAPI images are for reference. The 3D plots of the pixel intensity show peaks discrepant for intensity and location between the two antibodies on CD3+ follicular PD1+ cells. Scale bar 10 µm.
B comparison of PD1 antibodies UMAB197, UMAB199 and a goat anti PD1 polyclonal antibody on a sarcoma case (#14). The field is the same shown in Figure 4. The 3D plots of the pixel intensity show peaks discrepant for intensity and presence between the three antibodies. Scale bar 10 µm.

Supplementary references

1. Gendusa R, Scalia CR, Buscone S, et al: Elution of High Affinity (>10-9 KD) Antibodies from Tissue Sections: Clues to the Molecular Mechanism and Use in Sequential Immunostaining. J Histochem Cytochem 62:519-531, 2014
2. Cattoretti G, Bosisio FM, Marcelis L, et al: Multiple Iteractive Labeling by Antibody Neodeposition (MILAN) Protocol Exchange, 2018
3. Bolognesi MM, Manzoni M, Scalia CR, et al: Multiplex Staining by Sequential Immunostaining and Antibody Removal on Routine Tissue Sections. Journal of Histochemistry & Cytochemistry 65:431-444, 2017
4. Scalia CR, Boi G, Bolognesi MM, et al: Antigen Masking During Fixation and Embedding, Dissected. Journal of Histochemistry & Cytochemistry 65:5-20, 2017
5. Carpenter AE, Jones TR, Lamprecht MR, et al: CellProfiler: image analysis software for identifying and quantifying cell phenotypes. Genome Biol 7:R100, 2006
6. Schapiro D, Jackson HW, Raghuraman S, et al: histoCAT: analysis of cell phenotypes and interactions in multiplex image cytometry data. Nature methods 14:873-876, 2017
7. Scalia CR, Gendusa R, Basciu M, et al: Epitope Recognition in the Human-Pig Comparison Model on Fixed and Embedded Material. J Histochem Cytochem 63:805-822, 2015
8. Kleffel S, Posch C, Barthel SR, et al: Melanoma Cell-Intrinsic PD-1 Receptor Functions Promote Tumor Growth. Cell 162:1242-1256, 2015
9. Lo Russo G, Moro M, Sommariva M, et al: Antibody-Fc/FcR Interaction on Macrophages as a Mechanism for Hyperprogressive Disease in Non-Small Cell Lung Cancer Subsequent to PD-1/PD-L1 Blockade. Clin Cancer Res, 2018
10. Sconocchia G, Zlobec I, Lugli A, et al: Tumor infiltration by FcγRIII (CD16)+ myeloid cells is associated with improved survival in patients with colorectal carcinoma. International journal of cancer Journal international du cancer 128:2663-2672, 2011

11. Natali PG, Bigotti A, Nicotra MR, et al: Distribution of human Class I (HLA-A,B,C) histocompatibility antigens in normal and malignant tissues of nonlymphoid origin. Cancer Res 44:4679-4687, 1984

SUPPLEMENTARY DATA

Supplementary data can be downloaded at https://data.mendeley.com
Cattoretti, Giorgio (2019), “The immune landscape of uterine leiomyosarcomas”, Mendeley Data, v1
DOI: 10.17632/46r9hcvpbd.1
http://dx.doi.org/10.17632/46r9hcvpbd.1

1- A folder "Primary csv data, all cases" *Primary data for all cases, from HistoCAT (contains 21 Case_xx_Clustnames.xlsx and 21 case_xx.csv)*
2- A folder "Case14-heatmaps_plots" *tSNE plots and heatmaps for Case 14 (contains all the .png files)*
3- Case14-csv.xlsx *Primary data for Case 14 from HistoCAT*
4- Case14-images_mask.zip *All biomarker images and segmentation mask for Case 14*
5- Case_14_ClustNames.xlsx *Phenotypic assignment of the phenogroups for Case 14*
6- CellProfilerMask.cpproj *CellProfiler pipeline to segment a CD43, CD45, CD68 and CD163 composite image.*
7- SupplementalData-Manzoni.xlsx *An Excel file containing 1) Single Case data; 2) Primary Antibodies, exposure time, fluo channel, sequence; 3) Secondary Antibodies; 4) Complete phenocluster: phenotypic assignment of the phenoclusters obtained for each case*
8- leiomyosarcoma_k50.RData *Primary data from HistoCAT for all cases*
9- script_public.R *R script to produce tSNE plots, Phenograph plots, phenotypic reassignment and heatmaps for all cases*

Supplementary Table 1

Nine cell type classification of the inflammatory infiltrate.

**Cell type**	**Markers required**
Lymphocytes	CD45hi. T cells: CD3+, CD4+ or CD8+ GZMB/GNLY± ; NK cells: CD45+, CD3-, GZMB/GNLY+
Histiocytes	CD68 and CD163 plus any myeloid marker except CD16
Tumor-associated macrophages	Histiocytes CD16+
Phagocytes	CD68+ CD163-
Inflammatory monocytes	LYZ+ VISTA+ plus isolated myeloid markers (CD14, TIM3)
Monocytes-macrophages undefined	Any combination of lineage-restricted myeloid marker, except the combinations listed above.
Endothelium	CD34+
Unclassifiable	Absence of restricted lineage markers, CD3-, presence of promiscuous markers
Junk	Artifactual staining profile

NOTE: Myelomonocytic lineage restricted markers: CD14, CD64, CD68, CD163, LYZ. Promiscuous markers: CD4, CD16, CD32, S100AB, TIM3. Despite the fact that sparse CD16+ CD68+ macrophages are detected in 3 normal myometria, the abundance and consistency of this phenotype in leiomyosarcomas suggest to retain the term TAM for this subset of macrophages [10]. Endothelial cells, characterized by a prominent CD34 signal, were coopted by proximity in the CD43+45 mask because of encroaching lymphomonocytic cells.

SUPPLEMENTARY TABLE 2
Composition and phenotype of the inflammatory infiltrate.

Cell composition of the inflammatory infiltrate

	Relative values				Absolute values		
	**Mean**	**±SD**	**min-max**	**Pos/Total**	**Mean**	**±SD**	**min-max**
Total inflammatory cells (CD43, CD45, CD68, CD163+)					*7,701*	*6,178*	453 - 22188
Segmented % of total DAPI+	*29%*	*24%*	3% - 93%	21/21			
All MonoMacs	*64%*	*13%*	41% - 86%	21/21	*5,702*	*4,969*	204 - 18938
Histiocytes	*20%*	*17%*	1% - 74%	19/21	*1,433*	*1,838*	142 - 7442
Tumor-associated macrophages	*40%*	*13%*	16% - 54%	20/21	*3,197*	*2,933*	204 - 11087
Phagocytes	*16%*	*12%*	2% - 34%	7/21	*1,883*	*2,391*	86 - 7156
Inflammatory monocytes	*4%*	*2%*	1% - 9%	16/21	*292*	*209*	45 - 686
Monocytes-macrophages unclassified	*12%*	*12%*	0% - 47%	15/21	*715*	*544*	66 - 2006
T Lymphs	*13%*	*10%*	3% - 29%	20/21	*1,135*	*1,244*	54 - 4635
CD4	*6%*	*6%*	1% - 16%	11/21	*465*	*423*	92 - 1524
CD8	*10%*	*6%*	2% - 19%	12/21	*1,073*	*960*	54 - 2987
NK	*13%*	*17%*	1% - 63%	20/21	*116*	*88*	34 - 270
Endothelium	*6%*	*5%*	1% - 21%	20/21	*378*	*397*	95 - 1838
Junk	*4%*	*7%*	1% - 34%	12/21	*376*	*522*	31 - 1808
Unclassified	*5%*	*5%*	4% - 13%	13/21	*515*	*439*	112 - 1701

Cell phenotype of lymphoid and myelomonocytic subsets

		**Mean**	**±SD**	**min-max**	**Pos/Total**
CD39	on Lymphs	55%	20%	16% - 74%	7/21
CD69	on Lymphs	71%	29%	23% - 100%	12/21
PD1	on Lymphs	56%	20%	32% - 100%	9/21
TIM3	on Lymphs	46%	19%	14% - 74%	9/21
CD4	on Lymphs	30%	22%	6% - 77%	11/21
FOXP3	on CD4	69%	36%	17% - 100%	10/21
CD8	on Lymphs	62%	23%	22% - 100%	12/21
TCF7	on CD8	42%	18%	17% - 74%	8/21
NK	on Lymphs	9%	8%	3% - 23%	8/21
CD8 & NK*	on Lymphs	71%	49%	14% - 100%	3/21
TIM3	on Macrophages	*30%*	*19%*	4% - 75%	14/21
PDL1	on Macrophages	*20%*	*15%*	1% - 50%	13/21
PD1	on Macrophages	*21%*	*13%*	4% - 56%	16/21
VISTA	on Macrophages	*8%*	*7%*	2% - 25%	12/21
DR	on Macrophages	*24%*	*17%*	3% - 55%	16/21
CD83	on Macrophages	*38%*	*29%*	3% - 89%	17/21
B7H3	on Macrophages	*33%*	*24%*	4% - 76%	16/21

NOTES: * in some samples, CD8 and NK cells are clustered in a single cluster.

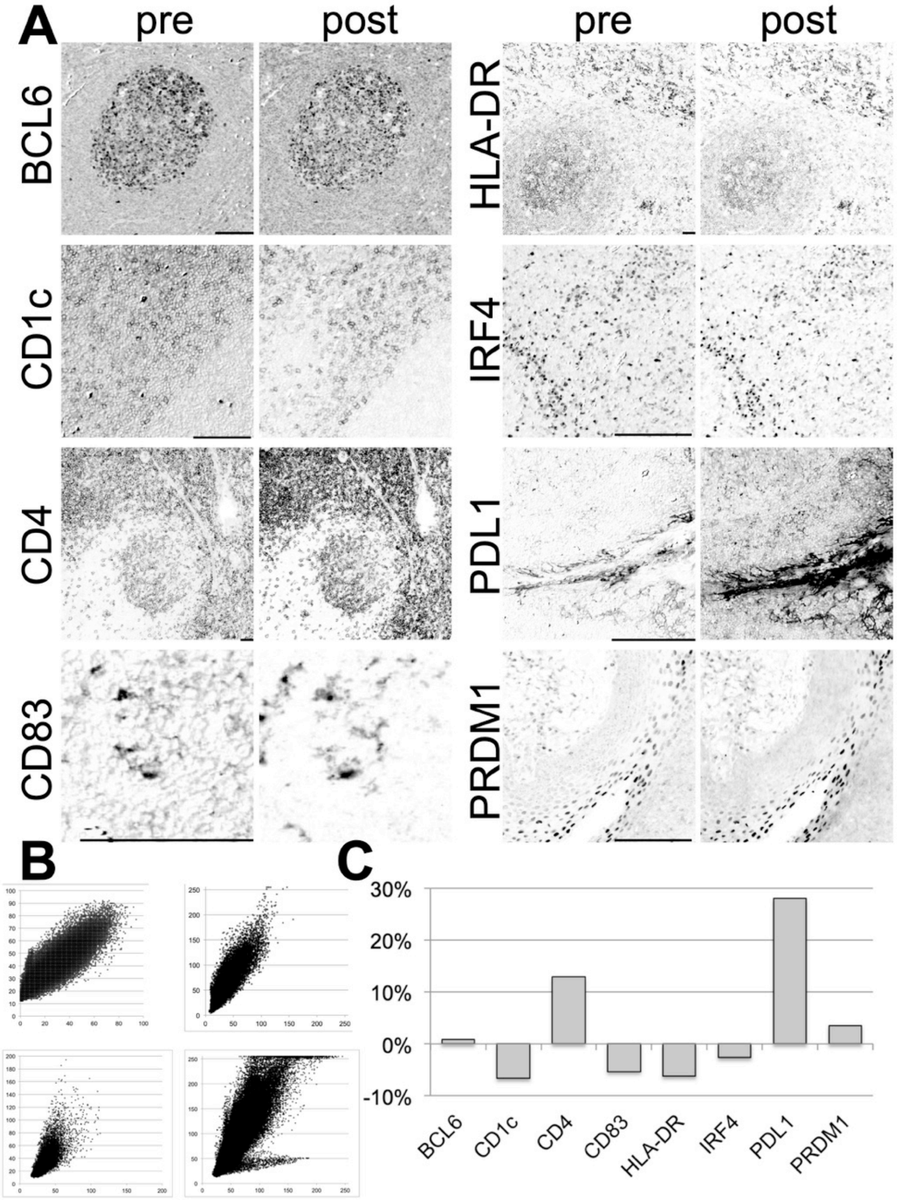

Supplementary Fig. 1 Effect of repeated rounds of staining and stripping on tissue immunogenicity.

A: Comparison of tonsil immunostaining in the first five rounds (pre) of a 27+ staining sequence and the same rounds performed after 27 cycles (post). CD4, CD83 and BCL6 repetition is after round 28, HLA-DR and CD1c after round 29, IRF4, PDL1 and PRDM1 after round 30. The antibody is shown at the left. **B**: pixel-by-pixel comparison of intensity before (x axis) and after (y axis) the staining cycles; clockwise from top left: DAPI, CD4, PDL1 on epithelia and on macrophages. No cell loss is shown by the DAPI comparison. Note that in the PDL1 images, AF was not subtracted and is represented by the horizontal species, not present after several cycles, as published before. **C**: The immunofluorescence quantification is represented as the channel variation from the pre to the post status, normalized for 256 channels. Scale bar : 100 μm. For details see supplementary methods.

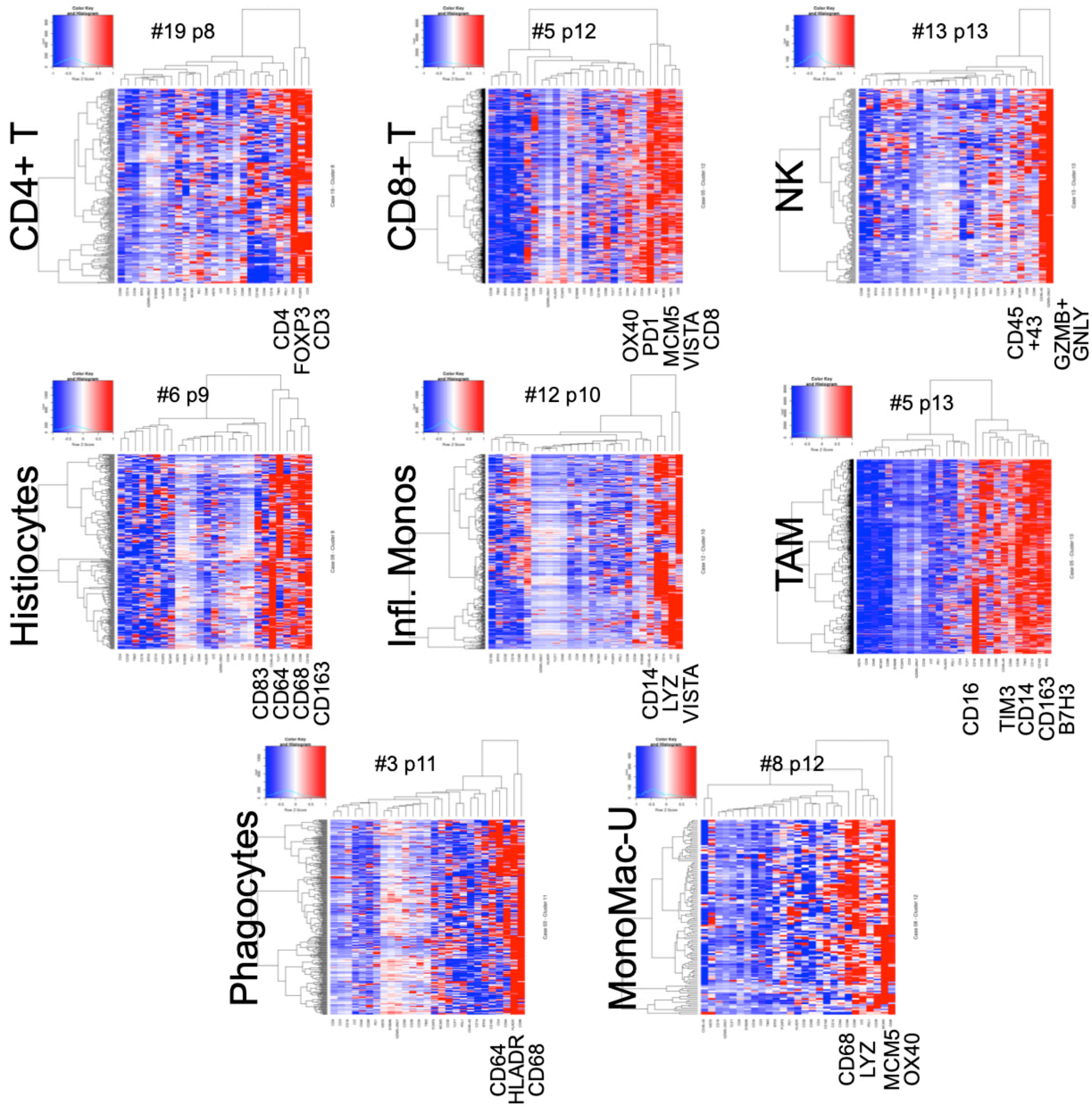

Supplementary Fig. 2 Heatmap representation of inflammatory cell subsets.
Heatmaps of selected phenoclusters, representative of the inflammatory cell subsets, as defined in Supplemental Table 1, are shown. The # indicated the case, the p indicate the phenocluster number of that case. Columns are the markers, rows are the cells composing that heatmap. Each heatmap has on the left the cell classification and at the bottom right the rightmost positive markers (red), enlarged. Negative values are blue, positive red, white intermediate.

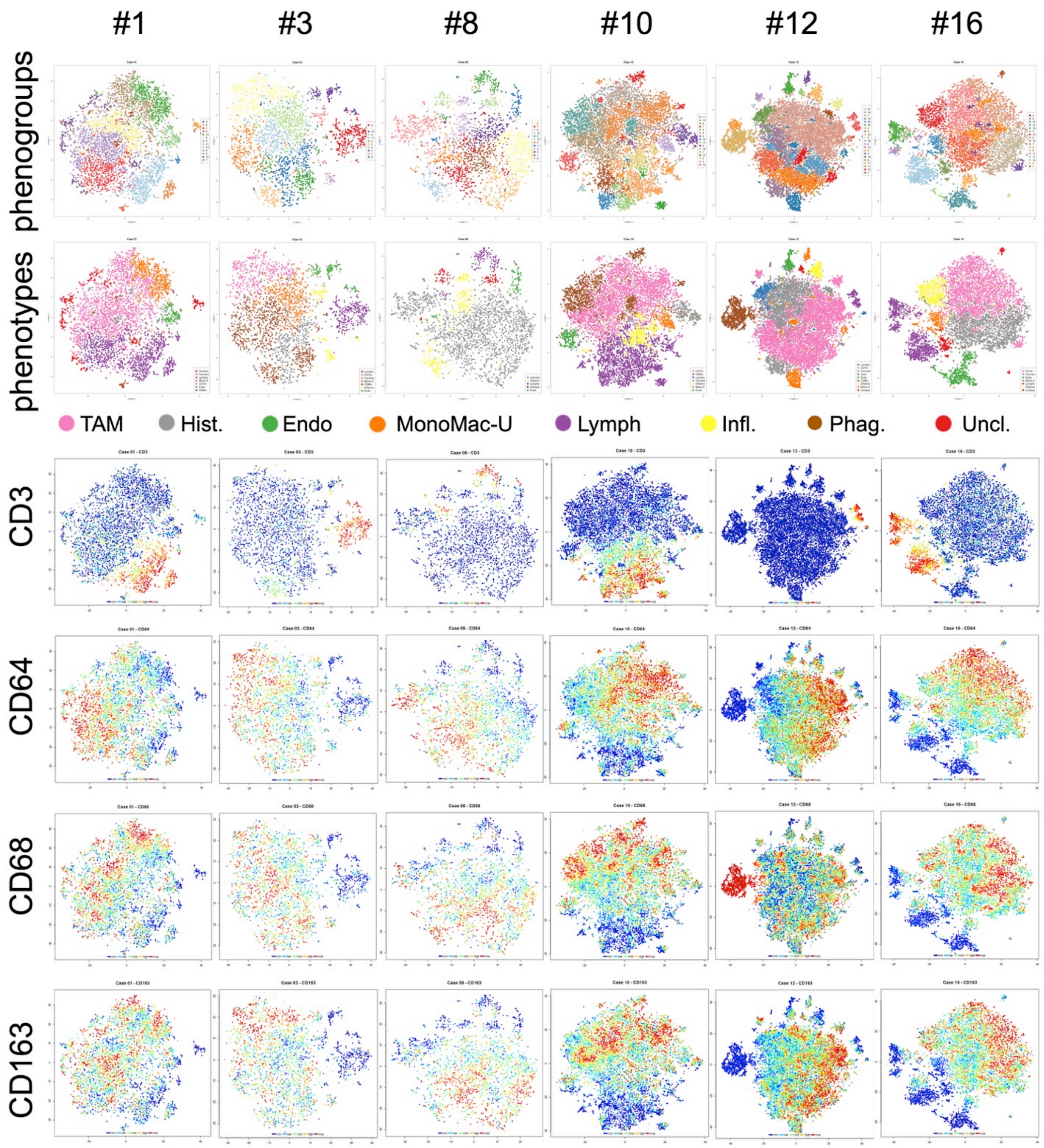

Supplementary Fig. 3 Classification of Phenogroups in Phenotypes.
Phenograph rendering of tSNE plots are shown in the top row for representative tumor samples (case N. on top). Below, the plots are redrawn with discrete phenotypic groups in color. In each column T cell (CD3) and Macrophage markers distribution on tSNE is represented. Case 8 represents non-tumoral myometrium.

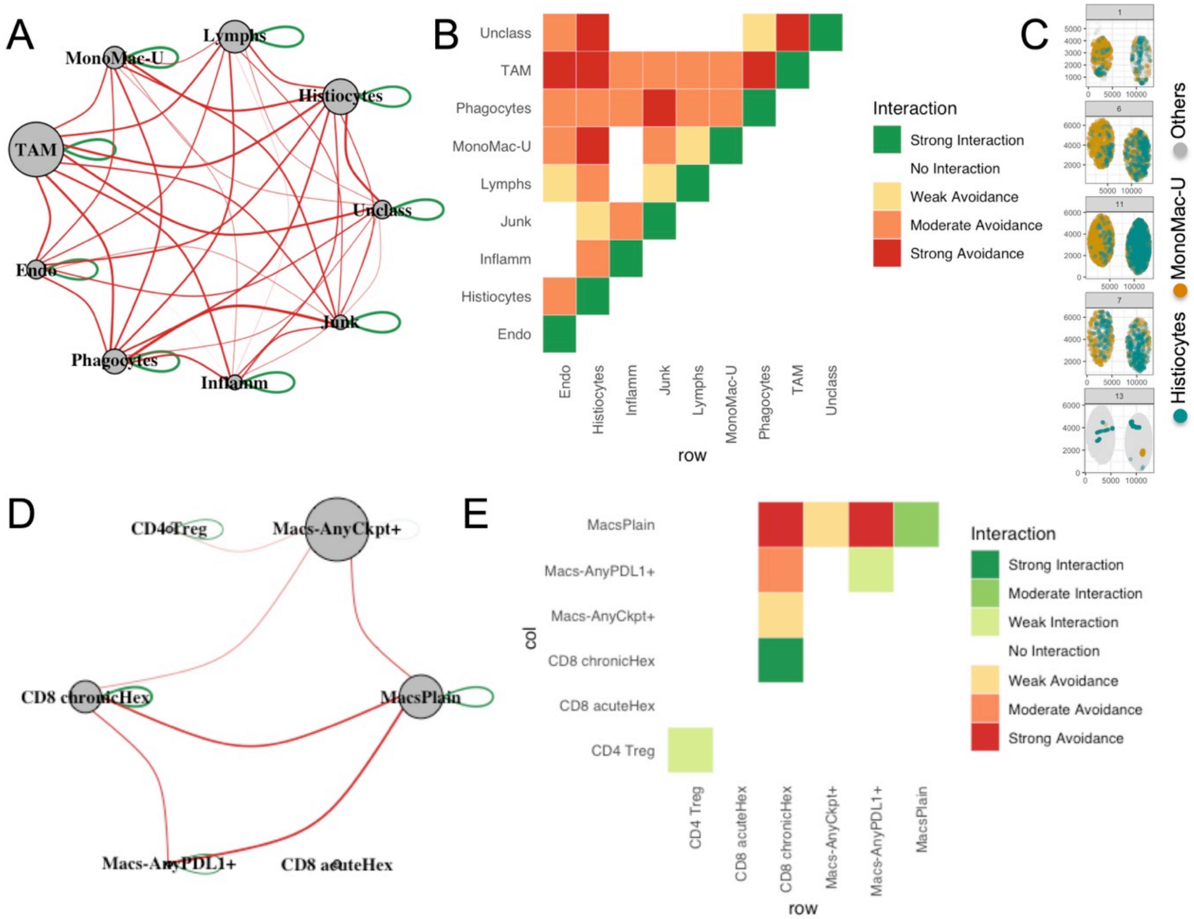

Supplementary Figure 4. Neighborhood analysis of the inflammatory infiltrate.
A social network of all the components of the inflammatory infiltrate.
B strength of the interactions of all the components of the inflammatory infiltrate. Note the strength of the homotypic interactions and the degree of avoidance for heterotypic interactions.
C five examples of the spatial distribution of two subsets with strong avoidance; each figure represents two TMA cores belonging to a case, which number is indicated on top of each image.
D social network of the relationship between T cell subsets and checkpoint molecules-bearing macrophages.
E strength of the interactions between T cell subsets and checkpoint molecules-bearing macrophages.

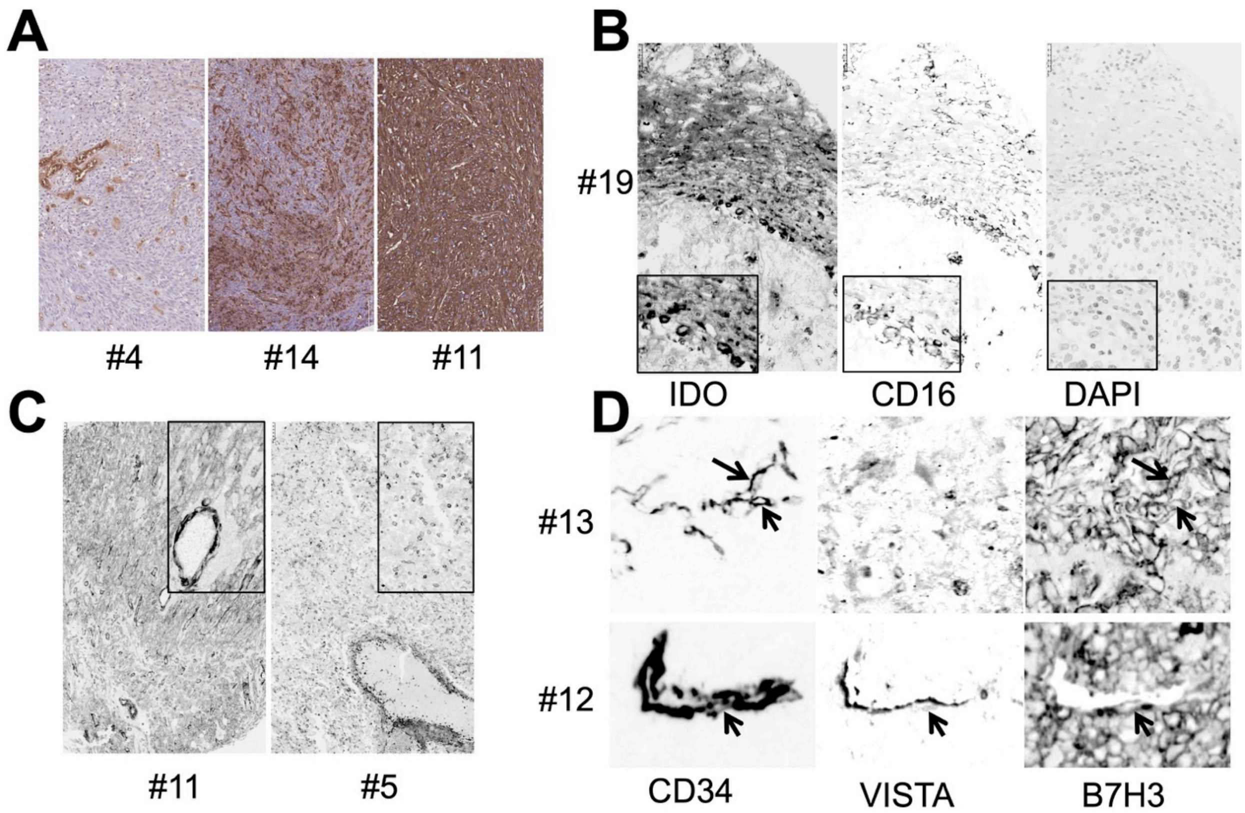

Supplementary Fig. 5. Phenotype of tumor and endothelial cells.

A HLA-ABC staining of representative cases. In case #4 only endothelia and scattered stromal cells are positive. Case #14 shows induced HLA expression in tumor cells close to inflammatory cells (lower half). Case #11 show strong constitutive expression in all tumor cells. IHC (DAB) in light microscopy. Note that Class I is reported absent from normal myometrium [11]. Scale bar: 100 μm.

B IDO expression on sarcoma and TAMs. Case #19. Tumor cells express IDO on part of the sarcoma cells and on TAMs. In the inset a group of CD16+ TAMs is strongly IDO+. All subtypes of macrophages express IDO (not shown). Inverted IF stain. Magnification 7x.

C AXL expression in sarcomas. AXL is expressed on vasculature-associated smooth muscle cells in both cases. In case #11 the tumor cells are diffusely membrane-positive (inset). In case #5 the sarcoma is negative; only smooth muscle cells and scattered histiocytes (inset) are positive. Inverted IF stain. Scale bar: 100 μm.

D Coexpression of checkpoint molecules on endothelial cells. Case #13 shows coexpression of B7H3 and CD34. Case #12 coexpress all three. Inverted IF stain. Magnification 7x.

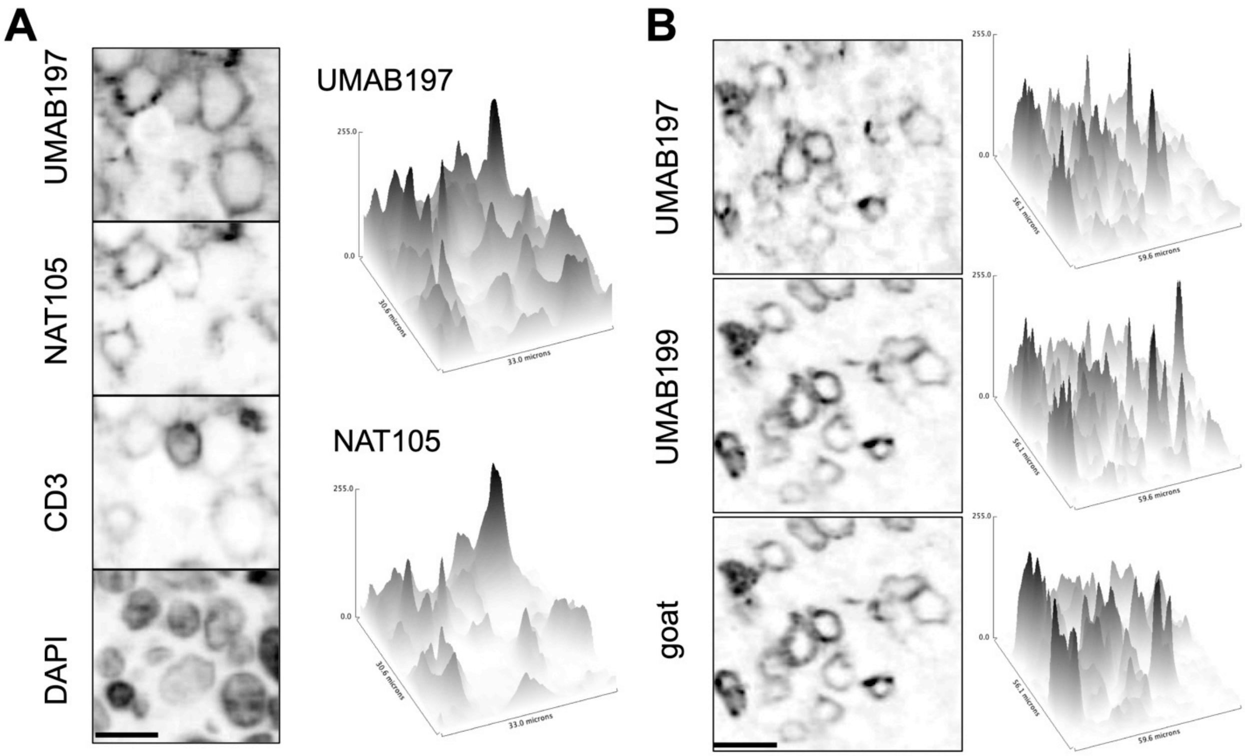

Supplementary Fig. 6. Identification of PD1 in tissue by antibody staining.
A comparison of PD1 UMAB197 and NAT105 staining on tonsil Germinal Center. CD3 and DAPI images are for reference. The 3D plots of the pixel intensity show peaks discrepant for intensity and location between the two antibodies on CD3+ follicular PD1+ cells. Scale bar 10 µm.
B comparison of PD1 antibodies UMAB197, UMAB199 and a goat anti PD1 polyclonal antibody on a sarcoma case (#14). The field is the same shown in Figure 4. The 3D plots of the pixel intensity show peaks discrepant for intensity and presence between the three antibodies. Scale bar 10 µm.
